# Mechanical and hydraulic properties of fractured Bentheim sandstone at different laboratory-simulated depths

**DOI:** 10.1038/s41598-026-61950-4

**Published:** 2026-07-14

**Authors:** Marco Fazio, Domenico C. G. Ravidà, Christian Ostertag-Henning, Martin Sauter

**Affiliations:** 1 LIAG Institute of Applied Geophysics, Stilleweg 2, 30655 Hannover, Germany; 2 Department of Applied Geology, University of Göttingen, Göttingen, Germany; 3 Department of Structural Geology and Geothermics, University of Göttingen, Göttingen, Germany; 4 Federal Institute for Geosciences and Natural Resources, Hannover, Germany

**Keywords:** Energy science and technology, Engineering, Solid Earth sciences

## Abstract

Understanding how rock properties change with depth is crucial for a variety of geoengineering applications. Even rocks that are homogenous at both micro and macro scales, such as Bentheim sandstone, lose this characteristic once fractured. While recent studies have shown how concomitant changes in stress, temperature and pore pressure affects the evolution of intact sample permeability at depths, an equivalent study on fractured material is missing. Therefore, by combining a multi-methodological approach consisting of rock deformation experiments simulating depth conditions up to 4 km, thin section analysis and fluid composition analysis of water samples, the evolution of permeability of fractured Bentheim sandstone is investigated in this study. Results suggests that fine particles produced by the fracturing and the movements along these fractures play a crucial role in permeability evolution. When these particles are removed, the fracture constitutes a preferential pathway and, together with the chemical processes occurring on the rock–fluid system, lead to a 3–7 times reduction in permeability followed by a complete recovery of it after a simulated burial and exhumation path. On the contrary, when these particles are still present within the fracture zone, they impede fluid flow. This causes a slightly reduction of permeability during the burial path followed by almost constant values of permeability throughout the exhumation path. These findings provide crucial information for georeservoir applications and the transfer of results from laboratory experiments to in situ conditions for a correct prediction of hydraulic properties.

## Introduction

European countries face a major challenge in their goal to achieve net-zero greenhouse gas emissions by 2050 in line with the Paris Agreement of 2016. Based on data from 2023 consumption, fossil fuels still hold the largest energetic share, on average equivalent to 38% for oil, 20% for natural gas, and about 9% for coal^1^. Nonetheless, the last two decades have witnessed a gradual increase in the utilisation of clean, renewable and sustainable energy sources to produce heat and electricity. Additionally, in the interest of mitigating, if not reversing, global warming, several new approaches have been developed to capture excesses of carbon dioxide (CO_2_) from the atmosphere^2,3^. Renewable resources such as geothermal energy and white hydrogen, alongside carbon capture and storage (CCS) processes and nuclear waste repository techniques, rely on a deep understanding of the geological, thermal, chemical, hydraulic, mechanical and physical properties of porous media in the subsurface (c.f.^4–7^). Among these properties, permeability likely plays the most crucial role as it defines how easily fluid can circulate through the reservoir porous rock.

Rocks at depth may contain fractures and faults, which in turn may create preferential pathways for fluids, thereby controlling the permeability of the medium^8,9^. Indeed, permeability is not directly controlled by porosity but rather by the degree of interconnection between pores^10^. Thus, the presence of a fracture network tends to increase this interconnection, leading to overall higher permeability^8^. For instance, Fortin et al.^11^ found that in basalts, permeability nearly doubles when comparing the intact rock to the same sample after shear failure. Similarly, Nara et al.^8^ show that permeability increases by 3.5 orders of magnitude and by more than 20 times in basalts samples with cracks induced by Brazilian tests and high-temperature thermal treatment, respectively. Conversely, in very highly porous and permeable rocks, such as Bentheim Sandstone, fractures can disturb the pre-existing pore-interconnectivity and size distribution of equant pores, ultimately triggering an overall reduction in permeability^12^.

With increasing depth, pressure and temperature conditions change, affecting not only the intact rock permeability (e.g.^13^), but also the fracture permeability. The first attempt to investigate the effects of elevated pressure and temperature conditions on fractured rocks was made by Nelson et al.^14^, who tested saw-cut Navajo sandstones. The authors discovered that permeability decreases non-linearly with confining pressure, whereas it exhibits a bell-shaped trend with increasing temperature: *k* initially increases due to increasing bulk volume but subsequently decreases due to the expansion of the mineral matrix, which reduces the available pore space. Although they could not test the effects of both pressure and temperature simultaneously, the authors suggested that the permeability of fractured sandstones would decrease non-linearly with depth. A comparable depth-dependent behavior was also postulated by Rutqvist^15^ based on experiments performed on both in situ blocks and ultra-large (1 × 1 m) cores of fractured granite.

Yasuhara et al.^16^ and to some extent Cheng et al.^17^ reported an opposite temperature-permeability behaviour than the one originally described by Nelson et al.^14^: both observed an initial reduction in *k* at lower temperatures, followed by an increase at higher temperatures. The described transition was attributed either to a shift between fracture aperture reduction and aperture widening caused by mineral redistribution^16^ or to a shift between pressure solution-dominated behavior to free-face dissolution processes^17^. Zhang et al.^18^ reported similar results, although the influence of temperature on permeability was largely disregarded. Cyclic loading experiments on thermally pre-treated fractured granite also show the role of temperature, which leads to thermal expansion of the mineral matrix and irreversible loss of permeability at elevated temperatures^19^

By far, the most studied parameter controlling the permeability is the stress field. Walsh^9^ showed that the cubic root permeability of a tension fracture in Barre granite, tested by Kranz et al.^20^, decreases logarithmically with effective pressure, according to the following model:

1 $$\sqrt[3]{k}=A-B\ln {p}_{eff}$$

where *k* is the permeability, *p*_*eff*_ is the effective pressure, and *A* and *B* are empirical constants. This model applies well at low effective pressures, when fluid-flow behaviour is controlled by macro-fractures, but it breaks down at higher pressures, where micro-fractures dominate the flow regime^8^. Conversely, Yang et al.^21^ found that the decrease in permeability with effective stress tested on fractured shales is best fitted by an exponential law. Multi-fractured basalt cores exhibit higher permeability than single-fractured samples. However, as the effective stress increases, the difference between these two cases vanishes due to the progressive closure of the fracture network^8^. Overall, increasing effective pressure promotes fracture closure, resulting in a permeability reduction (e.g.^19,22–25^^,^^26^^,^^27^).

The effect of changing pore pressure conditions on the permeability of fractured rocks has been investigated by a handful of studies. These works show that increasing fluid pressure counteracts the fracture closure, enhancing non-linearly the permeability of fractured granite^9,28^, and resin^29^.

Shearing along a fracture has multiple effects on permeability. On the one hand, a positive correlation has been reported for rock-fracture replicas made of resin^30^, fractured ice^31^ and fractured granite^32^, attributed to higher fracture heterogeneity, opening of existing cracks and the grain size of the cataclastic material, respectively. On the other hand, a negative correlation has been observed in fractured, highly porous sandstone^12^^,^^26^, where permeability decreases due to perturbations of the pore space. For sandstone with relatively low porosity (8.5%), low displacement levels and clay content in the order of 8%, fracture shearing does not significantly affect permeability, suggesting that this is matrix-dominated rather than fracture-dominated^33^. Moreover, using an innovative experimental setup in which the horizontal stress field is step-wise rotated, Fraser‐Harris et al.^29^ observed that the direction of shear (i.e. dextral vs. sinistral displacement), in combination with the fracture surface geometry, influences permeability, even under identical stress conditions.

In addition to shearing, also stress cycles on fractured samples affect permeability, therefore exhibiting a stress-path dependency. Multiple cycles induce irreversible stiffening and closure of the fracture surfaces leading to lower permeability levels at successive cycles^33,34^.

Nonetheless, despite the variety of studies attempting to explain the various controls on fracture development and fluid flow in porous media, no work to date has investigated how these variables interact when they are varied simultaneously to simulate real conditions at different depths in the Earth’s crust. Thus, the purpose of this study is to fill this knowledge gap by testing the permeability of shear-fractured Bentheim sandstone under systematic variations of key reservoir parameters, namely stress, temperature and pore pressure, both sequentially and simultaneously. Contrary to previous studies that focused on either saw-cut (e.g.^14,16^) or previously fractured via indirect tensile strength test samples (e.g.^8,34^), only a few studies^12,15,32,33^ dealt with shear-fractured samples containing comminuted material produced by failure and shearing. Additionally, this study took a step forward by implementing a multi-analytical approach that combines classical rock deformation testing with acoustic emission and ultrasonic monitoring, thin section analysis on post-test material and chemical analysis on fluids collected during testing. Such as an approach has never been employed before and the combination of this diverse dataset may shed light on the THMC coupled processes occurring at depths relevant for georeservoir characterization.

## Materials, equipment and method

### Experimental setup

As this study represents a continuation of the work done by Fazio et al.^13^, Bentheim Sandstones collected from the same quarry in Gildehaus (Germany), but originating from different blocks, were tested in the same internally heated, servo-controlled triaxial apparatus at the Laboratory of Experimental Hydro-Geomechanics (LEHG) at the University of Göttingen. In this study, however, all samples were cored with the axis parallel to the bedding layers, with a size of 100 × 250 mm. A total of eight samples were tested: BS18, BS19, BS21, BS22, BS23, BS24, BS25 and BS26. Given the homogeneity at the block scale of this material, we assumed density (*ρ*), porosity (*n*) and P-wave velocities (*v*_*p*_) values to be the same as those reported in Fazio et al.^13^: *n* = 24.37 ± 0.05%, *ρ* = 1987 ± 13 kg/m^3^, *v*_*p*_ = 2540 ± 104 m/s (parallel to bedding) and 2248 ± 97 m/s (perpendicular to bedding).

Compared to Fazio et al.^13^, 4 additional Acoustic Emission (AE) sensors were added to the AE sensor array, bringing the total number to 20 sensors, although during the experiment BS25 two sensors were faulty. The sensors were hosted in an engineered Nitrile jacket^35^, which embraced the rock sample and avoided the interaction between this and the confining medium.

The assumptions and the experimental procedure described in Fazio et al.^13^ were followed with two substantial differences:

1. Intact sample permeability has been measured at the initial conditions. This is followed by a shear deformation test (*σ*_1_ − *σ*_3_ = *σ*_*diff*_) leading to shear failure. After failure, *σ*_*diff*_ was lowered to the initial condition and the same experimental procedure as in Fazio et al.^13^ was applied to samples BS21, BS23, BS24 and BS25. While in BS21 and BS23 the starting simulated depth was 180 m, in BS24 and BS25 the starting simulated depth was 300 m. All other steps represented the same simulated conditions of 1, 2, 3 and 4 km. In all experiments permeability measurements were taken both during the burial and the exhumation path, apart for the measurement at the shallowest simulated depth during burial in BS24, where a leak through the engineered jacket was found.
2. Due to the failure, comminuted materials are mobilised, interfering with the pore fluid flow, increasing the viscosity of the fluid and obstructing both the pore channels and the piping of the apparatus. Therefore, experiments were conducted under either closed system or open system conditions. In the closed system, pore water was continuously recycled by refilling the empty upstream pump with water coming from the downstream pump after permeability measurements. In the open system, the empty upstream pump is refilled with fresh water while the water in the full downstream pump has been discharged.

For each experiment, we collected mechanical, hydraulic and temperature data, while AE data collection and ultrasonic monitoring (UT) were performed only in 2 experiments (BS21 and BS25) to retrieve the P-wave velocities of the material. An overview of the experimental conditions and techniques is shown in Table 1.

**Table 1:** Overview of the experimental conditions and techniques used for the tests and analysis on BS.

Sample	*σ*_*3*_ (MPa)	*p*_*p*_ (MPa)	*σ*_1_ (MPa)	*T* (°C)	AE array	*k* tests	System	Type	Thin sections	Chem. analysis
BS18	3–80	1–70	5–82	15–135	No	Yes	Closed	Seq	Yes	No
BS19	3	1.5	3.6	19	No	No	–	–	No	No
BS21	3–80	1.5–40	3.6–96	19–135	Yes	Yes	Closed	Simul	No	No
BS22	3	1.5	3.6	19	No	No	–	–	Yes	No
BS23	3–80	1.5–40	3.6–96	19–135	No	Yes	Open	Simul	Yes	Yes
BS24	5–80	2.5–40	6–96	20–135	No	Yes	Open	Simul	Yes	No
BS25	5–80	2.5–40	6–96	20–135	Yes	Yes	Open	Simul	No	No
BS26	5–80	1–70	6–81	20–135	No	Yes	Open	Seq	No	No

*σ*_3_: horizontal stress; *p*_*p*_: pore pressure; *σ*_1_: vertical stress; *k*: permeability; AE: Acoustic Emission; Seq: sequential; simul: simultaneous.

Among all the samples, BS18 and BS21 were brought to fracture in a closed-loop system, whereas the other samples were tested in open settings. A sequential stepwise increase and decrease of horizontal stress (*σ*_3_), pore pressure (*p*_*p*_) and temperature (*T*) was arbitrarily chosen for experiments on BS18 and BS26, while a simultaneous change of these three variables was selected for the remaining experiments. BS18 experienced a 1.5 cycle of stepwise increase and decrease of each variable, before the thermal treatment and a final decrease of first *p*_*p*_ and then *σ*_3_ after that. Contrary to the experiments performed in Fazio et al.^13^, here permeability has been measured also after the thermal treatment, as the other two variables were decreased. Instead, on BS26 *σ*_3_, and *p*_*p*_ were only increased once before the thermal treatment and decreased afterwards. Considering what has been observed in Fazio et al.^13^, we believe that this change in procedure will not compromise the comparison between the results in BS18 and BS26. Due to jacket rupture after shear failure, the experiments on sample BS19 and BS22 were aborted, and no measurements on fracture permeability (*k*_*frac*_) were conducted.

Signals were automatically picked and filtered based on the number of picked arrivals (NP ≥ 3) and average signal-to-noise ratio (SNR ≥ 3) of the 20 waveforms composing each AE event. This database allows us to filter out false events (e.g. electromagnetic noise) and obtain the AE event rate. The P-wave velocities are calculated at the lower, middle and upper parts of the samples by averaging 4 velocities measured along 2 orientations (north–south and east–west) and two directions of travel (e.g. transmitter 1 to receiver 11, transmitter 11 to receiver 1).

### Petrographic modes and fracture characteristics

Finally, to rule out compositional effects on the responses of the triaxial stimulation, detailed petrographic analyses were conducted on representative samples from each block. Thus, four plugs (BS18, BS22, BS23 and BS24) were sampled from fractured regions of the core cylinders, thin-sectioned, and examined at the Geoscience Centre of the University of Göttingen, Germany, using a Zeiss AXIO Imager A2m microscope equipped with a 5-megapixel Zeiss Axiocam 305 color camera. Compositions were determined through point counting (300 framework components) on 0.5 × 0.5 mm grid using the Gazzi–Dickinson method, and sandstones were classified based on the relative abundances of quartz (Q), feldspars (F), and lithic fragments (L)^36–40^. Interstitial features, such as cement, matrix and porosities, were also recorded when intercepted by the crosshair during point-counting. Grain sizes were determined by measuring the long axis of 100 grains on a point-count basis, whereas grain contacts and roundness were qualitatively assessed by visual comparison with standard charts^41,42^. Additionally, thin sections were used to examine some textural characteristics of the induced fractures, including width, presence of filling material, with associated grain sizes and sorting, and modifications in the intergranular porosity. The outcome of the petrographic point-count is included in the supplementary material.

### Fluid geochemical analysis

**Fig. 1:**
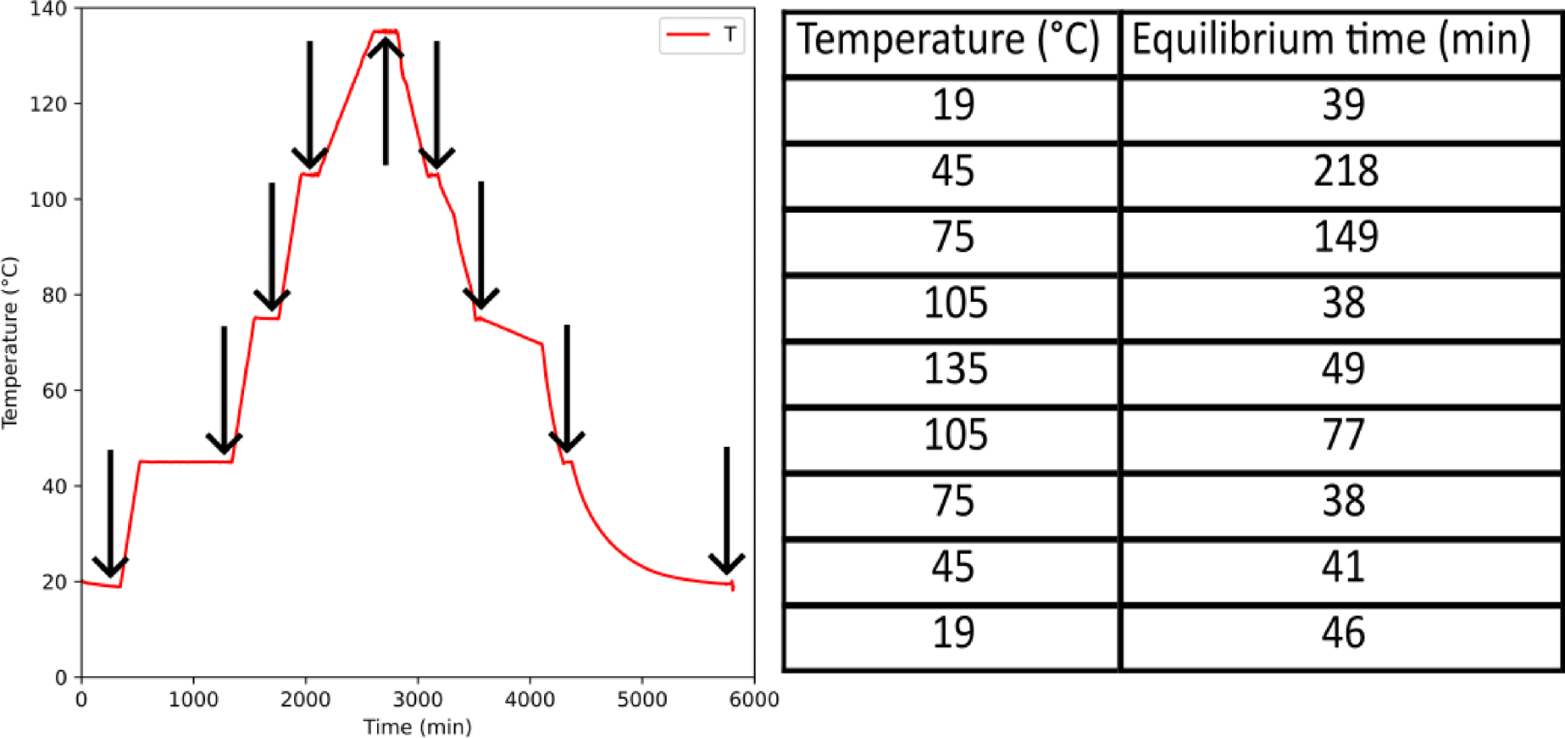
Temperature versus time plot during experiment BS23, with black arrows indicating the time of water sample collection through the fractured sample (left) and table showing the equilibrium time at each temperature step before water sample collection (right).

During experiment BS23, nine water samples were collected at each step representing a simulated depth, after temperature equilibration and before the permeability measurement (cf.^13^). This equilibrium time varied between 38 to 218 min, with six out of nine equilibrium times being between 38 and 49 min and a seventh equilibrium time being short of 80 min (Fig. 1).

The solution was collected into a 10 ml polypropylene vial, whilst the high-pressure pump connected to the bottom of the core maintained the pressure. In addition, both the feed water from the pump and the water flowing through the intact sample at the experiment start, hence at room temperature under confining pressure before fracturing, were sampled before the temperature increase program began. The water samples were centrifuged and filtered with a 0.2 µm cellulose acetate syringe filter before acidification with ultrapure HNO_3_. The concentrations of major and minor dissolved cations in the aqueous samples were analysed by ICP-OES (ARCOS III, SPECTRO Analytical Instruments GmbH).

## Results

### Thin section analysis

#### Sandstone composition and texture

The investigated Bentheim Sandstones are fine- to medium-grained and generally moderately sorted, with only sample BS23 exhibiting a poorly to moderately sorted texture. Grains are predominantly subrounded to subangular and typically display long or point contacts, while concavo-convex contacts are less common.

**Fig. 2:**
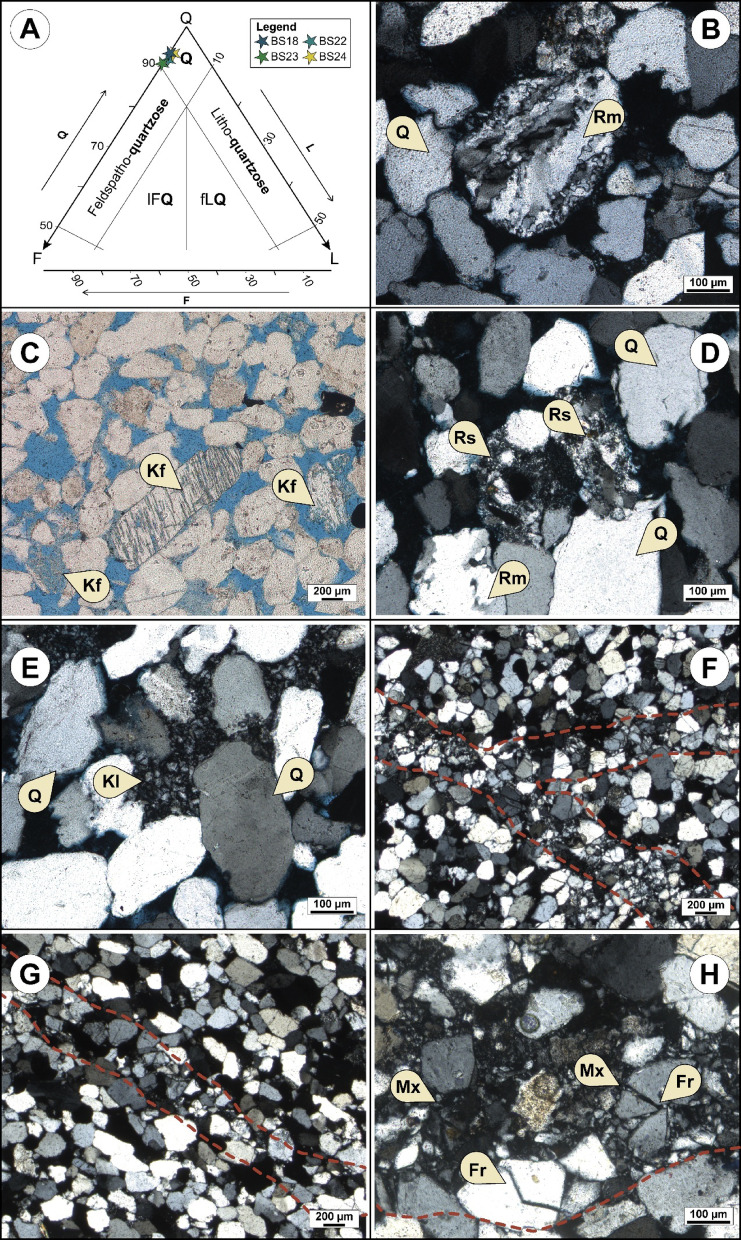
(**A**) Excerpt of the quartz (Q), feldspars (F), lithics (L) ternary diagram for sandstones^37^. The Q within the diagram marks the field of quartzose sandstones, whereas lFQ and fLQ indicate litho-feldspatho-quartzose and feldspatho-litho-quartzose sandstones, respectively. (**B**) Microphotograph showing quartz grains (Q) and gneissic rock fragments (Rm). (**C**) Partially dissolved K-feldspars (Kf) with evident secondary intragranular porosity. (**D**) Microphotograph showing quartz, metamorphic rock fragments and sandstone rock fragments (Rs). (**E**) Quartz grains and kaolinite cement (Kl). (**F**) Branching fracture zone in sample BS18. Notice the poor sorting within the fracture. (**G**) Fracture zone in sample BS23. (**H**) Detail of the fracture zone in sample BS18. Notice the rupture of grains (Fr) and the silty-size material filling the fracture area (Mx).

The examined samples classify as quartzose sandstones (Q_91–93_F_9–6_L_0–1_) (Fig. 2A), with quartz being the dominant detrital constituent, occurring primarily as monocrystalline grains (Fig. 2B) and occasionally as polycrystalline grains. Feldspars account for up to 9% of the detrital components and largely occur as K-feldspars, which are often partially to almost completely dissolved (Fig. 2C). The phaneritic assemblage constitutes less than 4% of the detrital components in all samples and mainly comprises gneiss fragments, with minor occurrences of schist, siltstone and arenitic lithic fragments (Fig. 2A,D). Micas occur rarely and no depositional matrix was identified. Nonetheless, detrital grains with a grain size < 63 µm intercepted by the crosshair were classified as siliciclastic matrix and generally represent less than 2.5% of the total sandstone composition.

Authigenic components occur in small amount—on average 3.7% of the total composition—and are mainly represented by kaolinite (Fig. 2E), commonly filling intergranular space or replacing feldspar grains. Rarely, replacive illite, authigenic quartz and Fe-oxide cements were identified. Additionally, samples BS18 and BS21 occasionally show brownish oil impregnations. Petrographic (or optical) porosity ranges from 13 to 20%, with most pores (10–13%) classified as intergranular porosity, whereas the remainder is secondary porosity resulting from the partial to complete dissolution processes (Fig. 2C) and rare grain fractures.

#### Fracture microscopic observation

Induced fractures were observed only in samples BS18 and BS23, where they occur as disturbance zones, occasionally branching, exhibiting highly fractured and disjointed grains embedded within otherwise undisturbed sandstone material (Fig. 2F–H). Their width varies from 250 to 460 μm in BS18 and between 370 and 430 μm in BS23.

Along their length, fracture dominantly appears as transgranular (cf.^43^), indicating that the stress propagated mostly through the grains resulting in a brittle deformation (Fig. 2H). Nonetheless, the intergranular propagation behavior is also observed.

Within the disturbed zones, rupture significantly alters the sandstone fabric, leading to a general decrease in grain size and sorting, along with an increase in both grain angularity and faceting. These changes ultimately reduce the textural maturity. This is particularly evident in sample BS18, where the fracture zone displays a bimodal grain-size infill: (1) very fine to fine angular grains, comprising approximately 61.7% of the total components; and (2) a siliciclastic matrix, likely consisting of smaller, angular, grain fragments, mostly silt-sized (< 63 µm), likely disaggregated during the hydraulic test e representing about 29.7% of the fracture infill (Fig. 2H). This finer grain fraction fills a substantial portion of the original intergranular porosity—observed in adjacent undisturbed regions—reducing to approximately 6%. Interestingly, it is also found within newly produced grain fractures. In contrast, the fracture zone in sample BS23 does not exhibit a significant fine-grained infill. Instead, grain size tends to decrease more uniformly, with a general increase in very fine- and fine-grained detritus at the expense of fine and medium sand, without the presence of a distinct silt-sized siliciclastic matrix significantly disturbing the intergranular porosity. No fracture zones were identified in samples BS22 and BS24, although occasional sparse clusters of fractured and disjointed grains were observed.

### Shear deformation and rock failure stage

The recorded maximum differential stress (*Max. σ*_*diff*_), i.e. the triaxial compressive strength, increases as the effective horizontal stress increases, ranging from 59.13 ± 2.38 MPa at *σ*_3*eff*_ = 1 MPa to 77.2 MPa at *σ*_3*eff*_ = 4 MPa (Table 2). The Young’s modulus (*E*) and the axial strain at failure (*ε*_*ax fail*_ in percentage) remains at similar level despite the changes in *σ*_3*eff*_. *E* and *ε*_*ax fail*_ are respectively 23.09 ± 0.70 GPa and 0.44 ± 0.04%.

**Table 2:** Experimental stress and pore-pressure conditions, mechanical and permeability results of the shear deformation test on BS.

Sample	*σ*_3_ (MPa)	*p*_*p*_ (MPa)	*σ*_3*eff*_ (MPa)	*Max. σ*_*diff*_ (MPa)	*E* (GPa)	*ε*_*axfail*_ (%)	*k*_*int*_ (mD)	*k*_*frac*_ (mD)
BS18	3	1	2	63.7	22.1	0.47	68	56
BS19	3	1.5	1.5	61.6	22.5	0.45	87	–
BS21	3	1.5	1.5	55.9	23.1	0.37	120	48
BS22	3	1.5	1.5	59.2	22.5	0.43	151	–
BS23	3	1.5	1.5	59.8	22.2	0.43	129	140
BS24	5	2.5	2.5	69.8	23.8	0.45	108	109
BS25	5	2.5	2.5	70	23.9	0.45	132	109
BS26	5	1	4	77.2	23.6	0.5	148	121

*σ*_3_: horizontal stress; *p*_*p*_: pore pressure; *σ*_3*eff*_: effective horizontal stress; Max. *σ*_*diff*_: differential stress at failure; *E*: Young’s Modulus; *ε*_*failure*_: strain at failure; *k*_*int*_: intact-sample permeability; *k*_*frac*_: fractured-sample permeability.

**Fig. 3:**
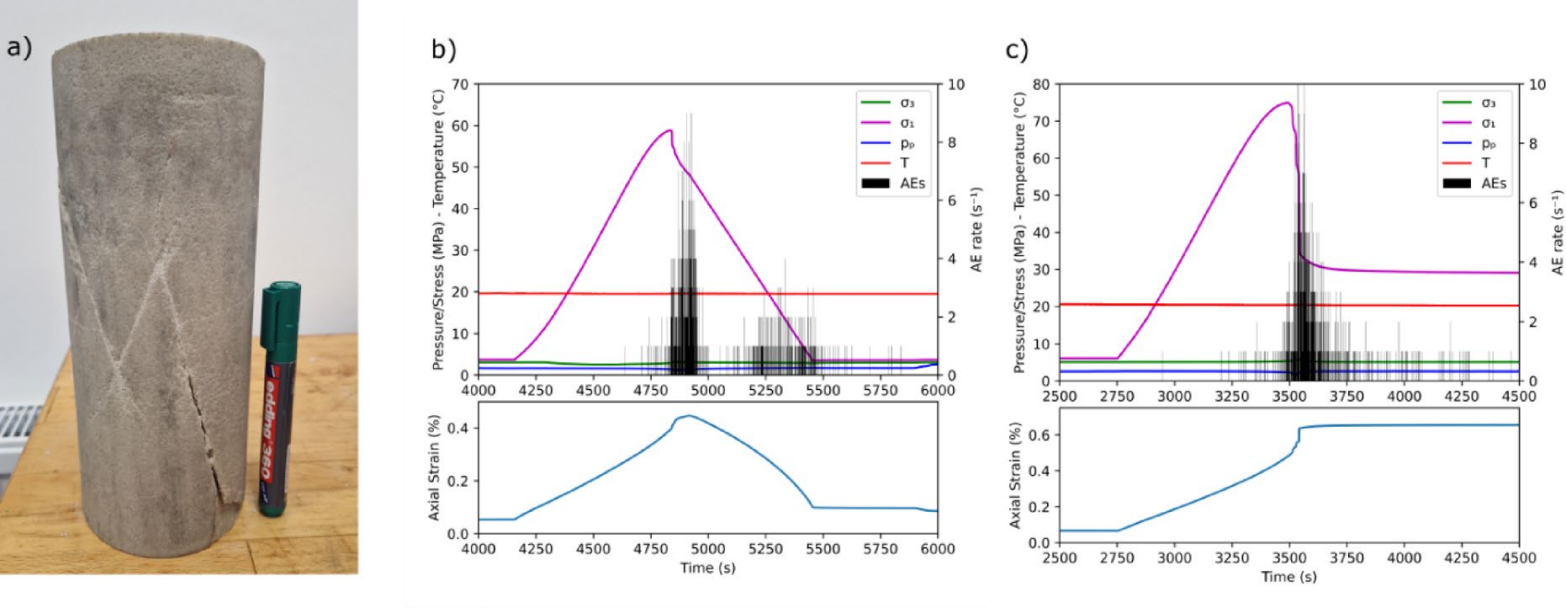
(**a**) BS23 post-failure sample, and time evolution of *σ*_1_, *σ*_3_, *p*_*p*_, *T* and AE rate (top) and axial strain (bottom) during the deformation stage on (**b**) sample BS21 and (**c**) sample BS25.

The BS23 post-failure sample, and the mechanical and AE data results of BS21 and BS25 are shown in Fig. 3. In both cases the highest AE rate, around 10 AEs per second, is observed immediately after sample failure, with a modest precursory swarm only seconds before failure. In sample BS21 (Fig. 3b), once failure occurred, the axial deformation was immediately reduced, bringing the stress level back down to the initial conditions. In sample BS25 (Fig. 3c) conversely, the axial deformation was only paused after failure allowing *σ*_1_ stabilized following the stress drop. This approach enabled us the measure the permeability of a fractured sample at critical stress conditions. Such permeability measurements were also performed on samples BS24 and BS26.

In terms of permeability, we observe an overall decrease from intact to fractured porous rock. In three cases, the permeability decreases by approximately 18% and in one case by 60%. In the remaining samples, it either remains at similar values or increases by 8.5%.

### Permeability evolution at different experimental conditions

#### Simulation of burial and exhumation on fractured rock

**Fig. 4:**
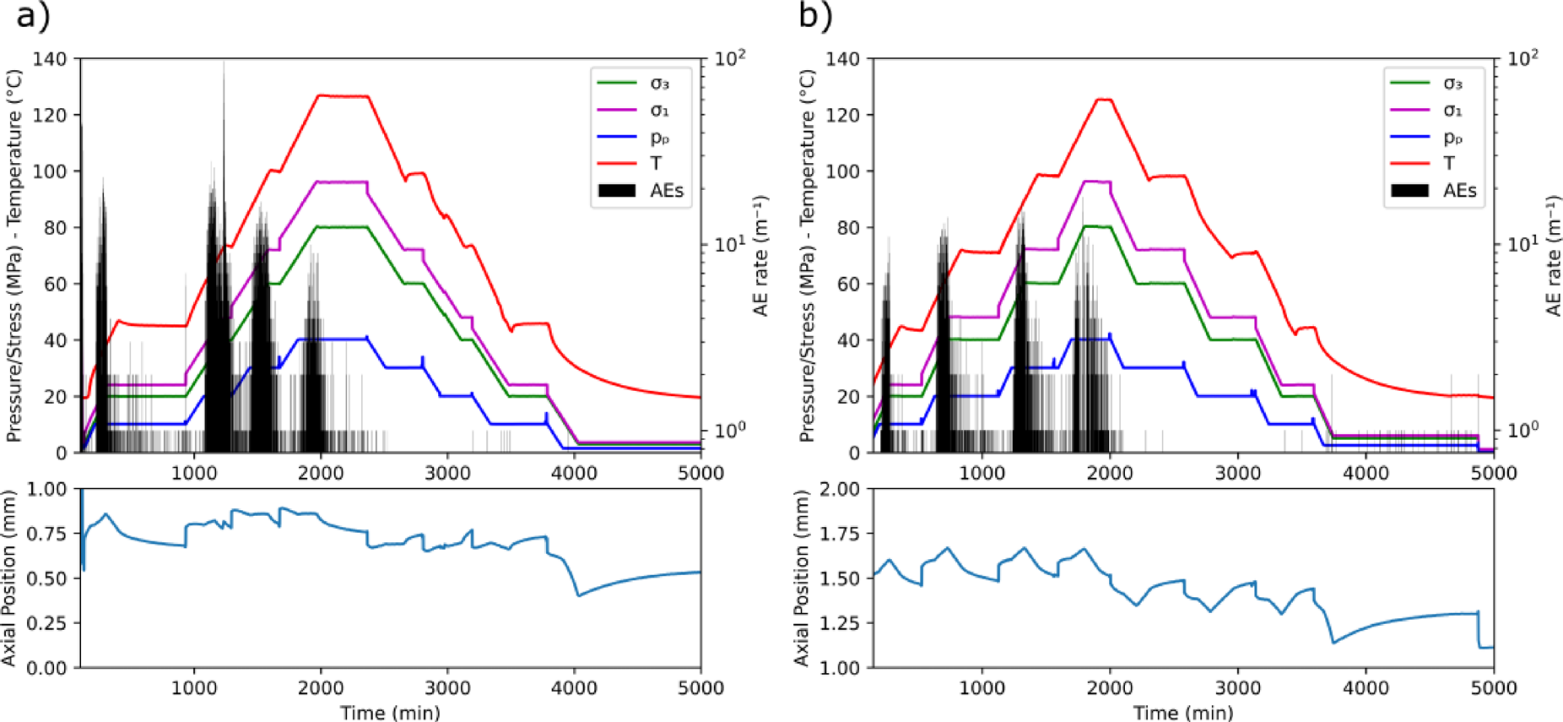
Time evolution of *σ*_1_, *σ*_3_, *p*_*p*_, *T* and AE rates (top) and axial position (bottom) during the simulated burial and exhumation stage on (**a**) sample BS21 and (**b**) sample BS25.

**Fig. 5:**
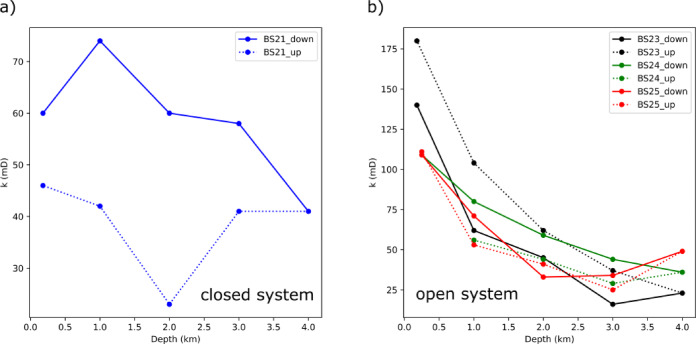
Variations of *k* with simulated depths during burial and exhumation on sample BS21 (**a**) and samples BS23, BS24 and BS25 (**b**). Burial paths in solid lines, exhumation paths in dotted lines.

The results of the permeability variations with depth are shown in Fig. 5. An overall decrease of *k*—from 60 to 41 mD at a simulated depth of 4 km—can be observed in experiment BS21 (Fig. 4a), which was run in a closed system. During simulated exhumation conditions, *k* maintains similar values to those reached at 4 km. All samples tested with the open system configuration show a similar permeability evolution with depth (Fig. 5b). Particularly, *k* decreases by a factor of a three- to seven during burial, reaching minimum values of 16 mD at 3 km for BS23, about 36 mD at 4 km for BS24 and between 33 and 34 mD at 2–3 km for BS25 Along the exhumation path, *k* retraces the burial trend in reverse progressively increasing and ultimately reaching values comparable or exceeding those at the initial conditions. In terms of AE activity (Fig. 4 and Table 3), experiments on samples BS21 and BS25 show a similar initial behaviour at shallow simulated depths, with AE rate between 13 and 25 events/min up to 2 km and a peak in filtered AEs during the transition between 1 and 2 km. Thereafter, in BS21 (Fig. 4a) the AE rate decreases to about 10 events/min, alongside the total number of filtered AEs during each transition down to a depth of 4 km. This is followed by a few events during stable conditions at this depth and even sparsely events during the whole exhumation path (maximum 9 from 4 to 3 km). In contrast, in BS25 (Fig. 4b) the AE rate slightly increases to 16 events/min while it remains at 3–4 events/min during stable conditions at 4 km. The maximum number of filtered AEs occurs during the transition at 3 km, although it is only slightly higher than the value observed during the previous step. During the initial and final steps on the exhumation path, several AEs were recorded, amounting to 22 and 38 in BS21 and BS25, respectively.

**Table 3:** Number of filtered AE events recorded at each step during experiments on BS21 and BS25.

Depth (km)	AEs in BS21	AEs in BS25
1	1059	333
2	2309	944
3	1444	969
4	671	716
3	9	22
2	1	0
1	5	2
0.18 or 0.3	1	38

**Fig. 6:**
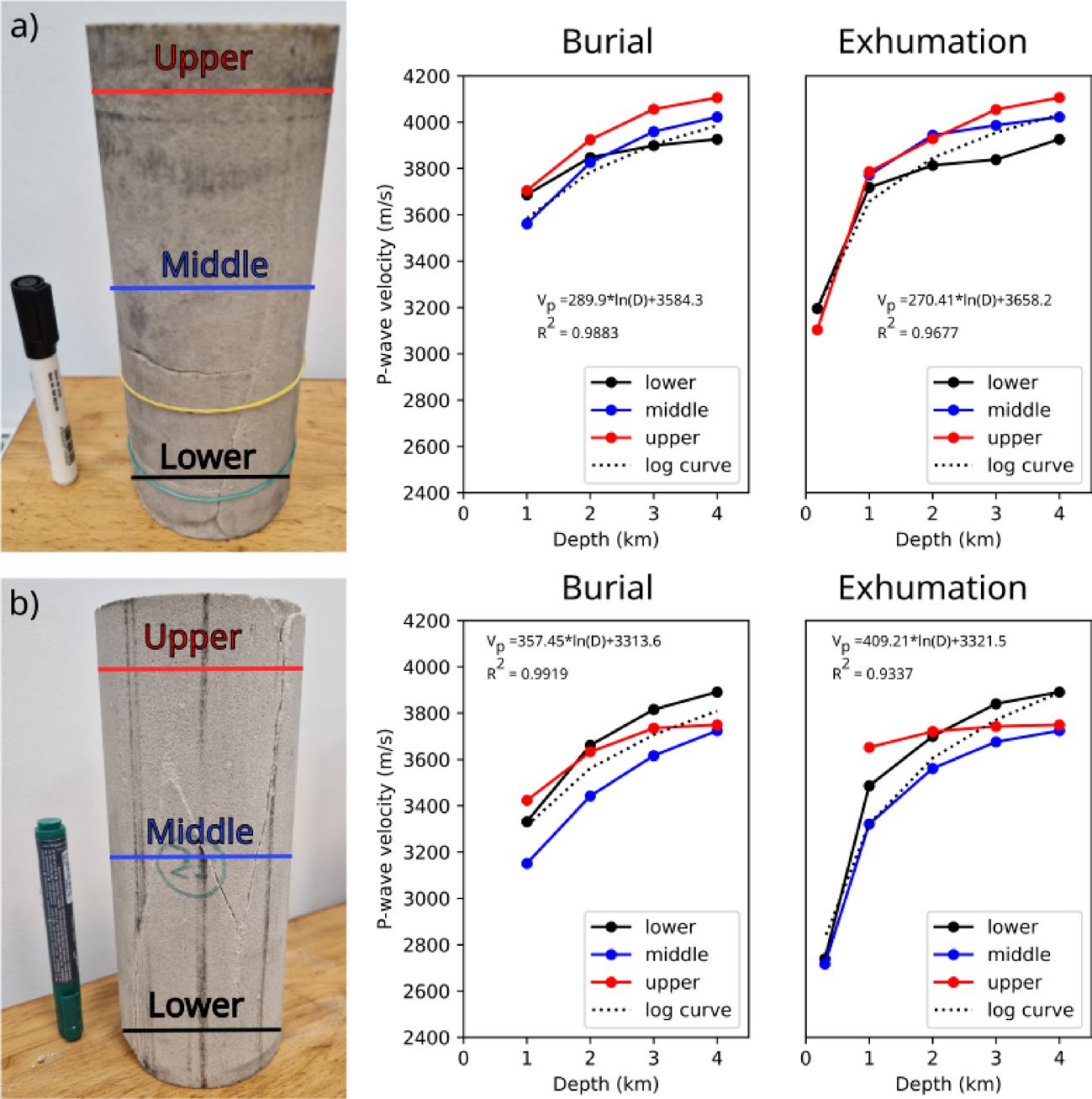
Variations of *v*_*p*_ with simulated depths during burial and exhumation on sample BS21 (**a**) and samples BS25 (**b**). Position of the lower, middle and upper part of the fractured samples, where velocity survey are performed, are shown on the left. In the centre and right plots the equations and the R^2^ coeffiecients refer to logarithmic curve modelled on the average *v*_*p*_ at each depth.

The P-wave velocities (*v*_*p*_) evolution of BS21 and BS25 samples is shown in Fig. 6. Although velocity at shallow depth during burial is not measured, both experiments reveal a similar pattern, with a logarithmic increase during burial and logarithmic decrease during exhumation (see log curve in dotted lines in Fig. 6). In BS21, all 3 averaged *v*_*p*_ are within 200 m/s from each other during burial and exhumation and both logarithmic curves, which are calculated by averaging all velocities at each depth, have similar scaling factor and a high R^2^ coefficient. In details, *v*_*p*_ increase from 3550 to 3700 m/s at a depth of 1 km to 3900–4100 m/s at 4 km during burial (Fig. 6a centre), whereas it decreases to 3700–3800 m/s at 1 km and to 3100–3200 m/s at a depth of 0.18 km during exhumation (Fig. 6a right).

In BS25 all *v*_*p*_ are within 300 m/s from each other, with different logarithmic scaling factor and lower R^2^ coefficients compared to those obtained in BS21. Throughout burial, *v*_*p*_ measured in BS25 remains systematically lower than in BS21, increasing from 3150 to 3400 m/s at 1 km depth to 3700–3900 m/s at 4 km (Fig. 6b centre). During exhumation, *v*_*p*_ decrease to 3300–3650 m/s at 1 km and to 2700 m/s at0.3 km depth (Fig. 6b right).

#### Sequential stepwise increase and decrease of ***σ***_3_, ***p***_***p***_ and ***T*** on fractured rock

**Fig. 7:**
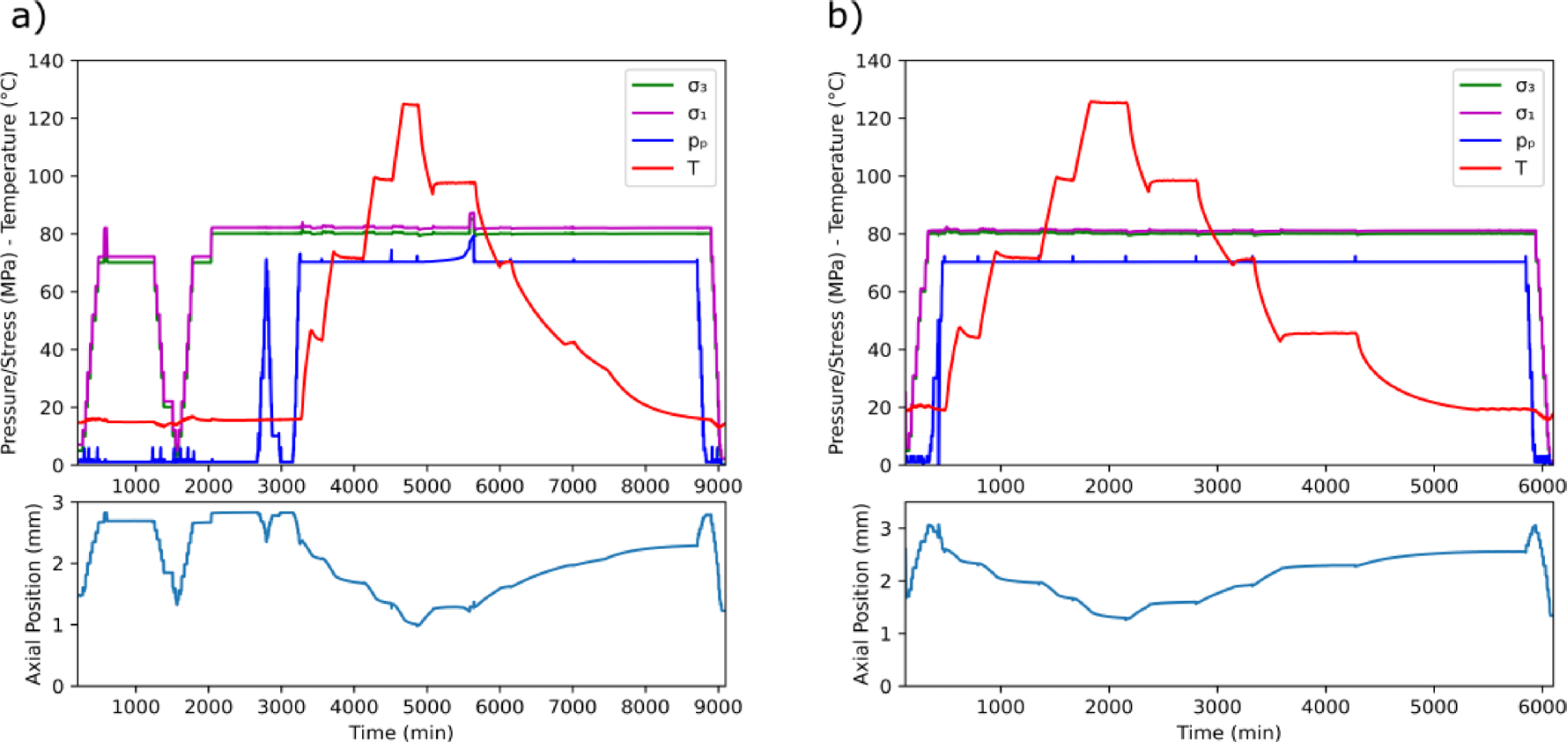
Time evolution of *σ*_1_, *σ*_3_, *p*_*p*_ and *T* (top) and axial position (bottom) during step-wise increase and decrease of individual variables on (**a**) sample BS18 and (**b**) sample BS26.

Figure 7 shows the experimental procedure applied for the experiments with sequential stepwise increase of individual variables. Additionally, during *σ*_3_ and *T* steps, changes in the pore space were calculated in the same way as in Fazio et al.^13^. However, since the starting porosity of the samples of the current studies was not determined, here we show the changes in the sample’s pore volume.

**Fig. 8:**
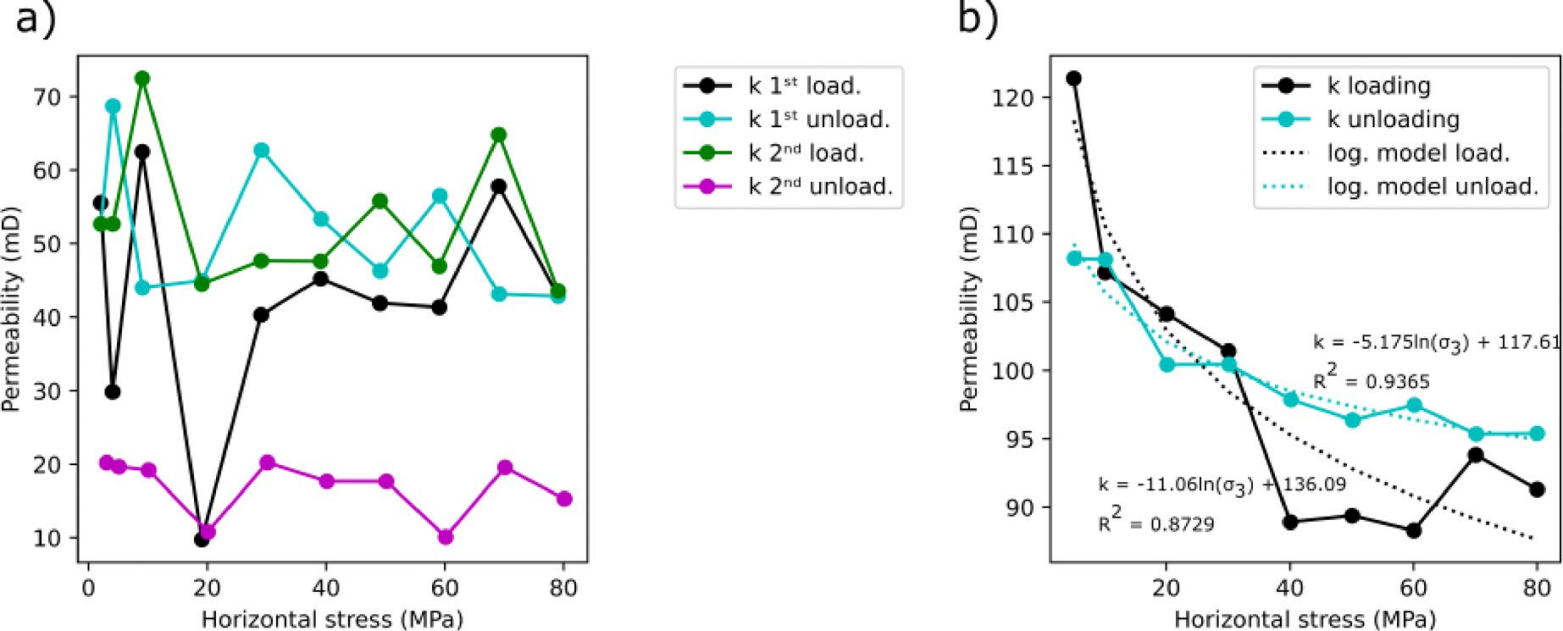
Variations of *k* with increasing and decreasing horizontal stress on (**a**) sample BS18 and (**b**) sample BS26.

During the experiment BS18, a non-monotonic, minor negative correlation of permeability with *σ*_3_ was observed, with values ranging between 53 and 72 mD at low stress levels and between 43 and 64 mD at higher stress levels. Following thermal treatment, permeability shows no trends and maintains values between 10 and 20 mD, more than 66% less than pre-treatment values (Fig. 8a). Permeability changes during experiment BS26 show a clearer logarithmic trend with *σ*_3_ variations, particularly at 5 < *σ*_3_ < 40 MPa. Before the thermal treatment, *k* decreases from 121 mD at low stress levels to around 90 mD at high stress levels, followed by an increase from 95 to 108 mD. Contrary to BS18, *k* values after thermal treatment are, in BS26, similar to those of pre-treatment conditions (Fig. 8b).

**Fig. 9:**
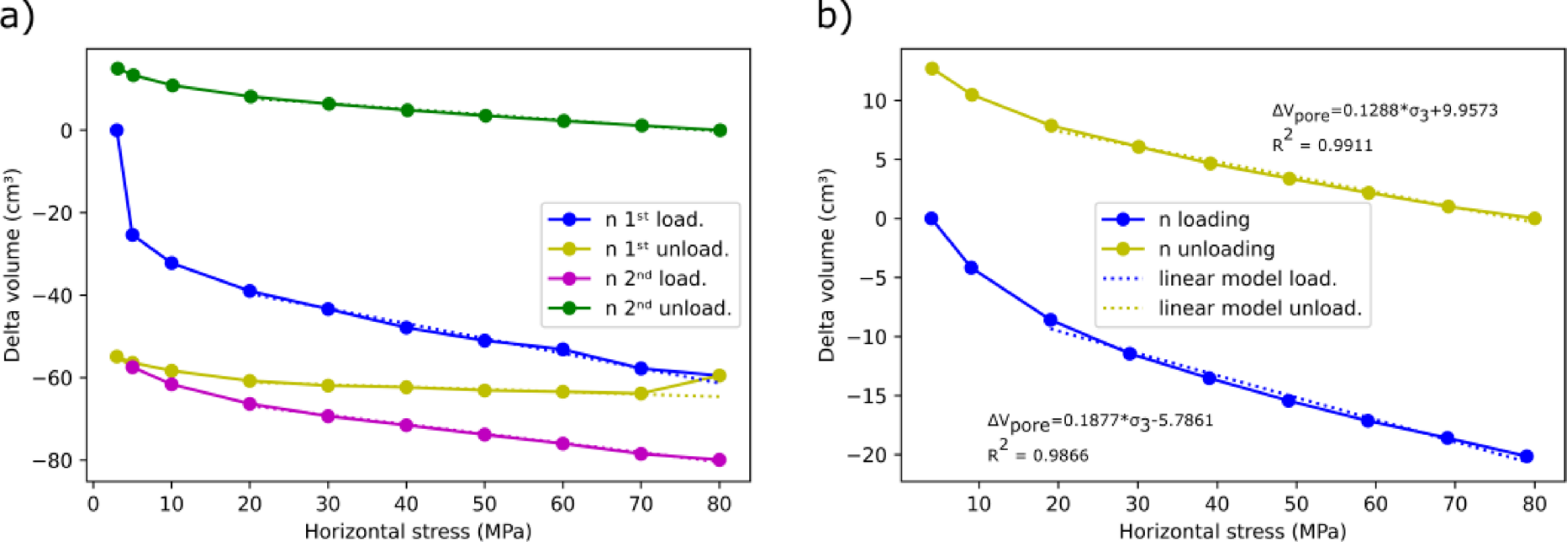
Variations of delta pore volume with increasing and decreasing horizontal stress on (**a**) sample BS18 and (**b**) sample BS26.

Changes in the pore volume are similar in both experiments with higher pore volume reduction at low horizontal stresses, although BS18 exhibits greater loss than BS26. Particularly, the largest, non-linear loss occurs until *σ*_3_ = 20 MPa, followed by a linear behaviour in all cases. Successive cycles, either pre- or post-thermal treatments, only show partial pore volume recovery. Sample BS18 exhibits a 55 cm^3^ pore volume loss during the first loading–unloading cycle, whereas the second cycle, halted by the pressurization and thermal cycles, show a pore volume loss of 10 cm^3^ (calculated considering the 25 cm^3^ lost during the 2^nd^ loading and the 15 cm^3^ gained during the 2^nd^ unloading, Fig. 9a). Conversely, sample BS26 lost 20 cm^3^ of pore volume during the loading phase and recovered 13 cm^3^ of it during the unloading phase, having a 7 cm^3^ net loss (Fig. 9b).

**Fig. 10:**
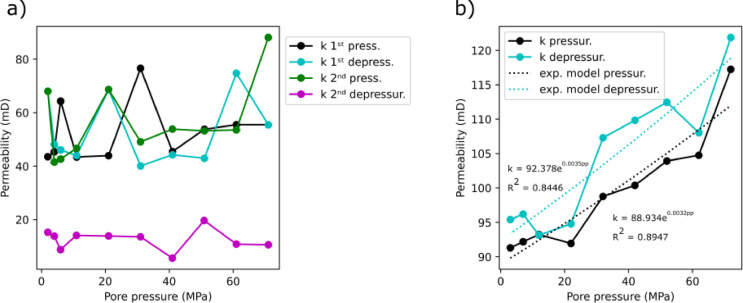
variations of *k* with increasing and decreasing pore pressure on (**a**) sample BS18 and (**b**) sample BS26.

Variations of *k* with *p*_*p*_ are less obvious. No trend can be seen in BS18, with *k* values measured before the thermal treatment being in the range of 40–88 mD, while after that the range is between 6 and 20 mD (Fig. 10a). Instead, an overall exponential trend showing a positive correlation, is visible in BS26, with values in the range of 91–122 mD both before and after the thermal treatment (Fig. 10b).

**Fig. 11:**
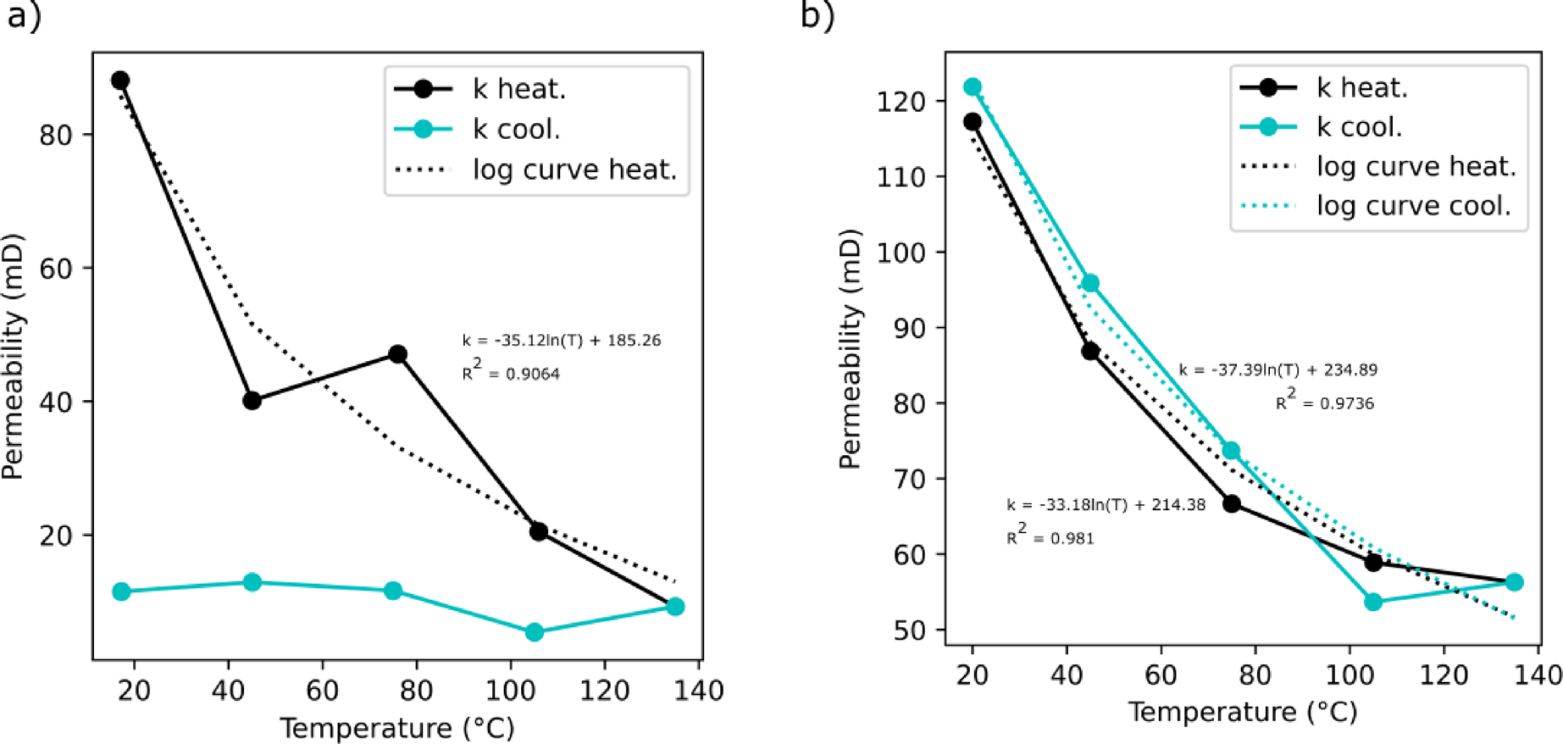
Variations of *k* with increasing and decreasing temperature on (**a**) sample BS18 and (**b**) sample BS26.

The effect of temperature on permeability is highlighted in both experiments by an inverse correlation: *k* decreases logarithmically as temperature increases, although this trend fits the observed data better in BS26. However, during the cooling the behaviour of *k* behaves differently between the two experiments. In BS18 there is no recovery as the temperature decreases to the initial values (Fig. 11a), whereas in BS26, the cooling path is almost specular to the heating one, showing a perfect recovery (Fig. 11b).

**Fig. 12:**
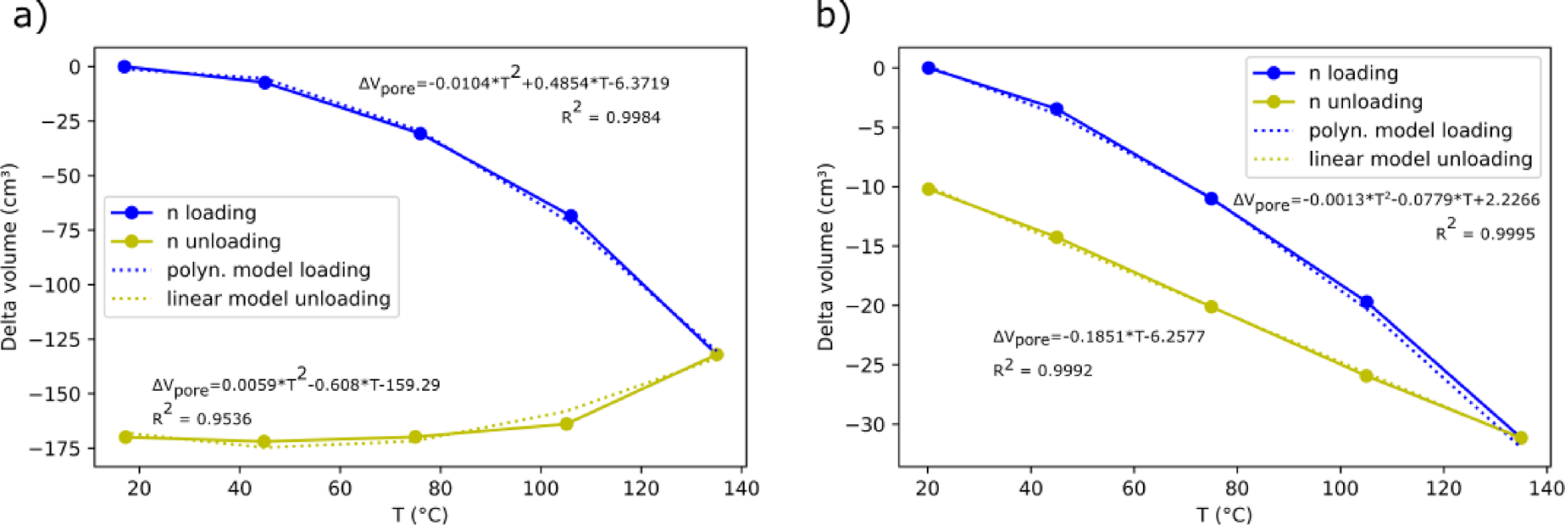
Variations of delta pore volume with increasing and decreasing temperature on (**a**) sample BS18 and (**b**) sample BS26.

During the heating and cooling stage, the variation in pore volume between the two experiments is significant. In BS18, there is 132 cm^3^ loss in pore volume, which followed a 2^nd^ degree polynomial, as the temperature increased, while another 2^nd^ degree polynomial fit the observed data during cooling with values of pore volume loss stabilizing around 170 cm^3^ (Fig. 12a). Similarly, in BS26 the pore volume loss during heating also followed a 2^nd^ degree polynomial but reaches a much smaller value at 135 °C (i.e. 31 cm^3^). Conversely to BS18, here there is a partial, linear recovery of pore volume during cooling, with a net pore volume loss of 10 cm^3^ (Fig. 12b).

### Fluid chemistry

**Fig. 13:**
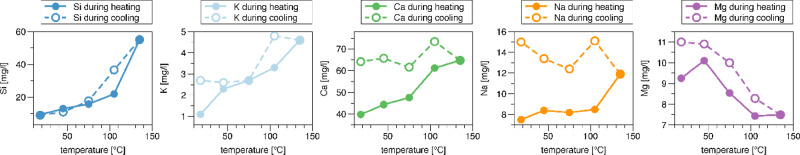
Concentrations of major dissolved ions at the heating and cooling stages during experiment BS23.

The results of the ICP-OES are shown in Fig. 13. Almost all measured elements increase in concentration in the pore fluid as the applied temperature rises. Nonetheless, different behaviours can be depicted. The concentration of Si and K increases significantly during heating, and shows a nearly reversible decline during cooling. In contrast, the abundance of Ca and Na increases with temperature and does not decrease significantly during cooling. The Mg shows a very different evolution, decreasing in concentration with raising temperature while increasing during cooling from 135 to 19 °C.

## Discussion

### Intact versus fractured rock permeability

As expected for highly porous rocks (> 15%^12^), we observe an overall permeability reduction when the sample failed in shear mode. Although this does not occur for every tested sample, the relative reduction far exceeds the relative increase in permeability in the only case of permeability enhancement after rock failure. Fractures are both visible at the macro (Fig. 6) and micro scale, the latter by the presence of bands with comminuted material (Fig. 2). This material is responsible for the permeability reduction as the newly formed smaller grains occupy previously empty pores, therefore reducing the pore connectivity.

Bentheim Sandstone is generally considered a homogeneous rock at different scales both in term of structure and mineralogy as well as an optimal reservoir (Fig. 2 and e.g.^44–47^). This is proved by the consistency of the mechanical properties (Table 2) across different samples and different stress conditions. The petrographic analyses conducted in this study also confirm these observations, showing that all the examined samples display homogeneous quartzose detrital compositions and initial moderate textural maturity^48^, as indicated by the dominance of monocrystalline quartz detrital grains, along with moderate sorting and rounding. These observations confidently rule out compositional variability as a controlling factor on the results of the triaxial simulations. Additionally, compaction and diagenesis appear to have had a minimal impact on the reservoir quality of these samples, as indicated by the consistently very low concentration of pore-filling cements (< 5% of the total composition) and the dominance of long or point grain contacts, suggesting limited compaction (c.f.^36,49,50^). Nonetheless, in the absence of precise constraints on the burial history of the examined samples, and given the evidence for dissolution processes (e.g., partially to completed dissolved feldspars), it is also possible that early diagenetic cements (e.g. carbonates) inhibited compaction during burial and were subsequently dissolved during exhumation (c.f.,^51^). Regardless of the process preserving intergranular porosity, all samples exhibit excellent petrophysical properties, with overall optical porosity—which is generally lower than the instrumental porosity (c.f.^52^)—ranging between 13 and 20%, and initial permeability between 68 and 151 mD. Nonetheless, good reservoir characteristics are lost within the induced fracture zones, where sandstone textural properties, such as sorting grain angularity and overall grain-size, are reduced, significantly affecting porosity. Indeed, although not directly comparable with instrumental measurements and despite the uncertainties associated with optical porosity assessment, intergranular porosity in the disturbed zone appears to have decreased by up to 40% relative to the values observed in the undisturbed zone. A similar behaviour was reported by Vajdova et al.^12^.

In our experiments the formation of a fracture system has 3 consequences: (1) it constitutes a structural feature (and possibly a preferential pathway for fluid flow), (2) it mobilizes grains and (3) it modifies the textural properties especially by creating finer grains. If these fine particles remain in the system, they can obstruct the fluid flow but also induce fast chemical reactions because of their very high reactive surface area^53^ at high temperature. This situation represents a real georeservoir scenario of a fractured rock with a damage zone and brocken material, which is formed during failure and shearing along the rock blocks.

If they are washed out in an open system, they do not impede the movement of fluids and the fracture surfaces, although roughs and tortuous, are free of comminuted material. This second scenario is similar to experimental conditions simulated in previous studies using pre-fractured samples (e.g.^8,34^), but these do not adequately represent real georeservoir conditions. The effect of these different behaviours is clearly observed in the evolution of permeability at depth between the open- and closed-loop experiments performed in this study.

### Open system scenario

**Fig. 14:**
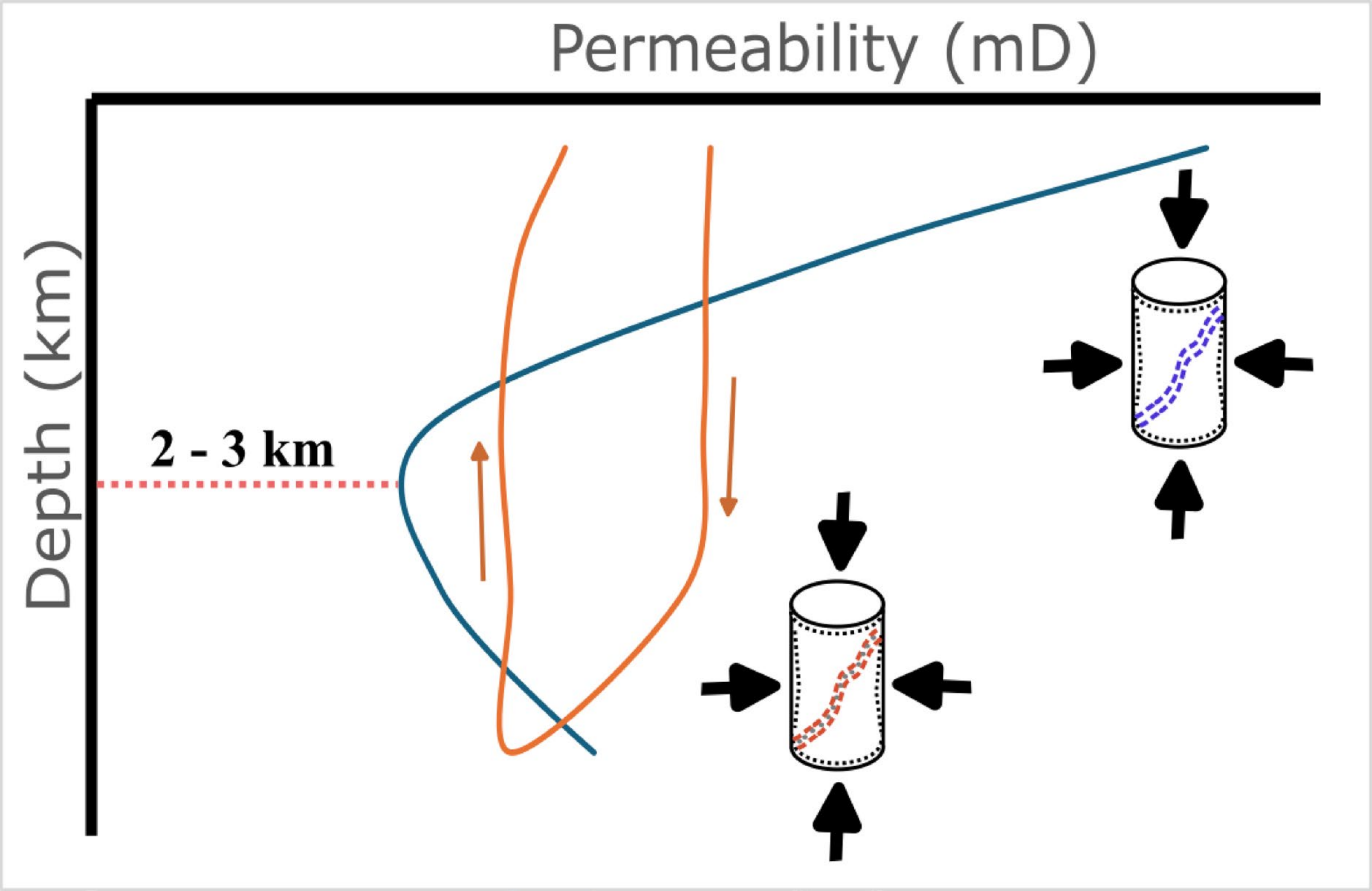
Sketch showing the evolution of fractured BS at depth during burial and exhumation for particle-free fractured sample (blue line) and particle-filled fractured sample (orange line).

Similar to results published in Fazio et al.^13^, permeability decreases with simulated depth, reaching a minimum between 2 and 4 km, and increasing again at higher depths (Fig. 14, blue line). During burial, pressure and temperature conditions tend to close not only the pore space, as for the case of intact rock, but also the fracture aperture. At higher depths, the presence of prolonged AE activity, particularly during the temperature increase, is a sign of microcracking which leads to higher permeability. In this context, the permeability is controlled by the still highly porous and permeable rock matrix rather than the fracture. During the exhumation, as stresses and temperature decreases, we observe a specular behaviour of permeability evolution, associated with AE activity at shallow conditions. We link this AE activity, greater than that observed for the intact sample in Fazio et al.^13^, combined with the higher value of permeability after the burial-exhumation cycle, to the movement of opening of the fracture. In addition, as more permeability values were measured and the upstream refilled with fresh water, the fracture was made free from the comminuted material allowing the fluid to flow through it. This is evident in sample BS23 (Fig. 2G), whose fracture zone shows almost no silt-sized matrix filling the intergranular pore space and a moderately uniform grain-size distribution. Under such conditions, at shallow levels the permeability of fracture Bentheim sandstone is affected by the presence of the disturbance zone, causing mechanical (fault movement) and chemical effects (dissolution and precipitation).

This phenomenon is clearly visible when the effect of individual variables on permeability is investigated. With changing horizontal stress, permeability follows a logarithmic change with it, with a better fit during the unloading phase (caused by a more efficient removal of crushed grains at later stages as the experiment progressed) associated to the closure of pore space and fracture aperture. This confirms the importance of a fracture system even on a highly porous and permeable sandstone. In fact, permeability behaviour on intact Bentheim sandstone show a negative relationship with horizontal stress only at low confinement as the pore space is preferentially closing along bedding layers^13^. Although the Walsh’s model^9^ was thought for a single fracture within a quasi-impermeable material, we can observe that a similar logarithmic behaviour characterizes fluid flow in a multi-fractured porous and permeable material such as the investigated sandstone. Additionally, this non linear behaviour fits well with non-linear fracture closure models postulated by Bandon-Bartis et al.^54^. In such conditions, particularly when fractures are free of particles and the logarithmic model yields higher R^2^ coefficient, permeability can be considered to be predominantly controlled by fracture network. However, retrieving the fractures aperture by applying the parallel-plate model^29,55^ is not feasible, as the rock sample hosts multiple fractures. Compared to the intact sample^13^, the influence of pore pressure on permeability of the fractured sample is more pronounced and follows an exponential law, with higher R^2^ coefficient obtained during the depressurization phase. This behaviour is consistent with the findings discussed in the previous paragraph. At higher water pressurization, permeability varies almost monotonous, either increasing or decreasing, indicating a greater efficiency of the pore pressure in opening the fracture zone, where the presence of comminuted material plays no role. The higher similarity of the coefficients and exponents of the exponential models found during the pressurization and depressurization, compared to the discrepancy between the constants of the logarithmic models during loading and unloading, as well as the specular behaviour of both pressurization and depressurization curve suggests that: (1) the subsequent thermal treatment had no permanent effect on the hydraulic properties of the rock and (2) the rock-fracture system remained unchanged between the onset of the pressurization and the end of the depressurization cycle, suggesting that the comminuted material was most likely completely removed from the system.

The effect of temperature on permeability is clear whether the rock is intact^13^ or fractured. The difference between the two cases is that in the fractured rock case we have a preferential pathway for fluid flood devoid of comminuted material. This causes a smaller decrease in permeability between the intact sample (− 72%) and the fractured sample (− 52%). By looking at the change in pore volume, we observed a net decrease in volume after the thermal treatment, which does not correspond to a net decrease in permeability after the thermal treatment. This information, combined with quasi-identical permeability–temperature curves and similar coefficients of the logarithmic model for both heating and cooling path, suggest that, although the pore space has irreversibly change, the fracture zone did not. Therefore, while the permeability evolution at depth in this scenario and in the intact rock case^13^ are very similar, the fractured case shows a full recovery or even an increase in permeability after the burial and a clear correlation between individual variables and permeability. This observation suggests that the permeability at depth is in fact mainly controlled by the particle-free fracture system.

The effect of the release of fine particles can be seen from the changes in fluid compositions as the fractured was brought to simulated burial and exhumation conditions. While none of the element compositions can reflect the change in permeability, as expected, they however shed lights to some chemical processes as pressure and temperature are simultaneously changed in an open system. The changes in fluid composition in experiment BS23 clearly document rapid fluid-rock reactions: the increase of the concentrations of almost all elements with temperature points to mineral dissolution reactions, and perhaps ion-exchange processes at the surfaces of the minor clay mineral component. The only ion with a decrease in concentration with increasing temperature is magnesium, which points to an early oversaturation and precipitation of a secondary, Mg-bearing mineral phase with increasing concentrations of e.g. Si. During the cooling stage the behaviour of the ions markedly differ: whereas Si and K nearly go back to the initial concentrations at the equivalent temperature during the heating stages—and hence invoke rapid attainment of fluid–rock equilibria by secondary precipitation during cooling, the concentrations of Ca and Na remain relatively stable without a significant decrease during cooling. The increase of Mg during cooling might be due to ion exchange processes with increased concentrations of Na on mineral surfaces. The lack of reprecipitation of Ca and Na is co-responsible (the other being the presence of a fracture system, see below) of the return to equal or higher level of permeability after the exhumation path as the solid material does not contain these precipitated elements. Additionally, as the fluid is now enriched in these dissolved elements, the viscosity of the fluid is higher compared to the initial fluid at the beginning of the burial path. This means that the calculated permeability at the end of the experiment has been underestimated due to the assumption of constant viscosity at same pressure and temperature conditions. For a refined interpretation of dissolution and precipitation reactions and possible attainment of equilibria, time resolved sampling at the different plateaus is needed, which is planned in future experiments.

### Closed system scenario

In a closed system scenario, where the silty-size comminuted material is still present within the fracture zone (Fig. 2F), the variability of the permeability under changing pressure and temperature conditions as similar to those observed by e.g. Kluge et al.^33^ and Vajdova et al.^12^. Rather than being flushed out, finer, silt-sized materials, produced by the fragmentation of detrital grains, continues to recirculate in suspension within the closed-loop fluid system and, after each cycle, accumulates and becomes trapped in the fracture zone, reducing the permeability of an originally highly permeable rock matrix. This would also explain why silt-sized matrix is found within newly produced grain fractures.

In fact, although at the macro-scale the samples tested at open system conditions and the samples tested at closed system conditions are both structurally (Fig. 6), texturally and compositionally (Fig. 2A) similar, their pore space volume is significantly different, particularly after the thermal treatment (Fig. 12). During the burial path and with increasing horizontal stress and pore pressure, permeability evolution is similar to that one observed in intact samples^13^, because the hydraulic conductivity is controlled by the deformed but still very permeable rock matrix rather than the silt-filled fracture zone. This can be also seen in pore volume change: in all cases (intact, filled fractured, empty fracture) there is a linear decrease in porosity/pore volume change after horizontal stress > 20 MPa and in both fractured scenarios, pore volume decreases following a 2nd degree polynomial trend. However, due to the presence of infill and loose material, there is a greater loss in both permeability and pore volume change in the closed system scenario than in the open system scenario.

The fractured rock under closed system conditions underwent significant modifications to its pore space and hydraulic properties at high temperature, not caused by mechanical effects as the elastic velocities do not differentiate substantially when comparing both burial and exhumation paths. In fact, after thermal treatment we observed a negligible change across all measured permeabilities after the maximum reached temperature both when variables were changed simultaneously and when they were changed sequentially, indicating the permeability did not recover during exhumation (Fig. 14, orange line).

We infer that the particle-filled fracture act as a physical barrier for the fluid flow, with comminuted material disrupting irreversibly the interconnectivity of the pore space. This constitutes the dominant controlling mechanism explaining the permeability evolution at depth.

### Sample inter-variability

Once fractured, Bentheim sandstone loses its structural homogeneity and different samples may produce variable results. Nonetheless, all the three samples tested in open system conditions with simultaneous change of variables (namely BS23, BS24 and BS25) show a remarkably similar permeability evolution at simulated depth. This suggests that the fracture process combined with the removal of comminuted material is not enough to cause a significant inter-sample variability in relatively large sample with highly homogeneous intact rock matrix. Different experimental conditions were instead simulated for the two samples tested in closed loop settings. Here the presence of comminuted material within the fracture may increase inter-sample variability as trapping rates and dynamics likely vary according to the distribution and tortuosity of the fracture network, as well as the initial fabric characteristics of the samples (e.g. grain size, sorting, matrix content). Additionally, compared to open-system conditions, fluid recirculation likely promotes dissolution and precipitation reactions, as solute concentrations vary over time. However, these two experiments, BS18 and BS21, produce coherent and correlated results that corroborate our overall discussion and interpretation.

## Conclusions

In this study we investigated the effect of a fracture system on the permeability of Bentheim Sandstone under pressure and temperature conditions representative of different depths. To do so we apply a multi-methodological approach consisting of (1) mechanical and hydraulic data from triaxial apparatus experiments, (2) mineralogic and microstructural data from thin section analyses and (3) fluid chemical composition from water samples collected during triaxial experiments.

When compared to previous experiments on the same rock in its intact status, these new results show the importance of the fracture system that, when devoid of fine particles, can maintain a high permeability similar to that of the intact permeability. On the contrary, when these fine particles are still present, they obstruct the fluid flow resulting in a permeability 2–3 times lower than the free fine particles case. Even so the overall behaviour of the permeability with depth between intact and fractured rock (in an open system scenario) is similar, the fracture tends to have a main role in controlling the permeability as seen when variables are individually investigated.

In addition, the evolution of fluid composition provides strong evidence for significant changes in pore-fluid chemistry within the experimental system, closely linked to the imposed temperature stepwise decrease. This highlights that the presence of a fracture network promotes both mechanical and chemical processes, whose complex interplay affects the permeability evolution at depth. In particular, fluid composition data, in combination with rock deformation experiments, highlight the potential to study more rapid geochemical reactions of mineral dissolution or reprecipitation and their effects on petrophysical and hydraulic properties in future experiments.

Finally, our results show the importance of studying fractured material at representative conditions as georeservoir rocks at depth are not ubiquitously intact and how the interaction of multiple variables, investigated through a multi-methodological approach, affects the hydraulic properties of such material.

## Data Availability

The datasets used and/or analyzed during the current study are available from the corresponding author upon reasonable request.
